# 17α-Hydroxyprogesterone Caproate Inhibits Cytokine Production via Suppression of NF-κB Activation

**DOI:** 10.3389/fphar.2022.831315

**Published:** 2022-03-07

**Authors:** Tao Hu, Chengjifu Tang, Sydney Stern, Luan Yang, Tom Du

**Affiliations:** ^1^ Evergreen Therapeutics, Inc., Bethesda, MD, United States; ^2^ Department of Pharmaceutical Sciences, University of Maryland School of Pharmacy, Baltimore, MD, United States

**Keywords:** cytokine release syndrome, 17α-hydroxyprogesterone caproate, COVID-19, SARS-CoV-2, NF-κB

## Abstract

Cytokine release syndrome (CRS) is one of the leading causes of morbidity and mortality in COVID-19 patients with elevated levels of circulating cytokines contributing to various clinical symptoms. Favorable control of CRS represents a promising and effective strategy to mitigate the clinical outcomes of hospitalized patients with moderate to severe pneumonia. Using *in vivo* cytokine release assay in human peripheral blood mononuclear cell (PBMC)-engrafted immunodeficient mice, we reported that 17α-hydroxyprogesterone caproate (17-OHPC), a synthetic progestogen, exhibited significant inhibition of OKT-3-stimulated production of numerous cytokines including TNF-α, IFN-γ, IL-2, IL-4, IL-6, IL-10, and GM-CSF. Furthermore, 17-OHPC inhibited *in vitro* production of IFN-γ, IL-1β, IL-2, IL-6, and IL-10 in human PBMCs stimulated with OKT3, while exhibiting down-regulation of the mRNA levels of TNF-α, IFN-γ, IL-2, IL-6, and IL-10. Using the same human PBMCs, additional stimulators anti-CD28 antibody or PHA treatments led to substantial cytokine production, which was also attenuated by 17-OHPC. OKT3-stimulated phosphorylation of IκBα and nuclear translocation of NF-κB p65 in human PBMCs were also reversed by 17-OHPC, suggesting its inhibition on NF-κB signaling in immune cells. Taken together, this work reported both *in vivo* and *in vitro* inhibition of cytokine production by 17-OHPC, presumably by virtue of its suppression of NF-κB signaling. These findings provide pharmacological evidence to support the potential application of 17-OHPC in treating CRS associated with COVID-19.

## Introduction

Cytokine release syndrome (CRS) is a life-threatening systemic inflammatory response involving elevated levels of circulating cytokines, leading to morbidity and mortality in patients with coronavirus disease 2019 (COVID-19), a global pandemic caused by infection of severe acute respiratory syndrome coronavirus 2 (SARS-CoV-2) (Que et al., 2021). The plasma levels of various inflammatory cytokines and chemokines such as interleukin (IL)‐1β, IL-2, IL-6, IL-7, and IL-10, tumor necrosis factor (TNF)-α, interferon (IFN)‐γ, granulocyte–macrophage colony stimulating factor (GM-CSF), chemokine C-C motif ligand (CCL)2, CCL3, and chemokine C-X-C motif ligand (CXCL)10 were elevated in COVID-19 patients. These elevations have been associated with severity of disease, worsening outcomes, and contribute to clinical symptoms including fever, coagulation, pneumonia, acute respiratory distress syndrome (ARDS), and multiorgan failure (Wilson et al., 2020). Considering the mounting evidence demonstrating the strong association between CRS and the clinical manifestations of COVID-19, favorable control of CRS is believed to be beneficial to the clinical outcomes of patients, in particular those with moderate to severe pneumonia.

Several therapeutic agents that suppress immune response and cytokine production have been tested clinically in the treatment of COVID-19, including anakinra, tocilizumab, baricitinib, etanercept, emapalumab, glucocorticoids, hydroxychloroquine, chloroquine, and more (Que et al., 2021). Those drugs target diverse molecular signals involved in CRS, such as IL-1 signaling, IL-6 signaling, TNF-α signaling, IFN-γ signaling, and Janus kinase-signal transducer and activator of transcription (JAK-STAT) signaling, with some of them exhibiting promising clinical benefits (Kim et al., 2021). For instance, multi-regional clinical trials have shown that tocilizumab, an IL-6 receptor monoclonal antibody used to treat CRS caused by chimeric antigen receptor (CAR)-T cell therapy, led to a reduction in hospitalization time as well as reduction in the progression to mechanical ventilation or death in COVID-19 patients (RECOVERY Collaborative Group, 2021; Salama et al., 2021). In June 2021, the U.S. FDA issued an emergency use authorization (EUA) for tocilizumab for the treatment of certain hospitalized adults and pediatric patients with SARS-CoV-2 infection. Albeit with observed beneficial effects in clinics, none of these therapies have been approved by regulatory agencies for COVID-19 treatment. Therefore, research and development of potential therapeutics targeting CRS in COVID-19 patients are highly desirable in the pharmaceutical industry.

Of note, COVID-19 exhibited differences in severity and mortality between sexes, male patients had almost three times the odds of requiring intensive treatment unit admission, and higher odds of death compared to females (Gerber et al., 2009), indicating the potential application of sex hormones in the pursuit of therapies for COVID-19. 17α-hydroxyprogesterone caproate (17-OHPC) is a synthetic progestogen that has been approved to reduce the risk of preterm birth in pregnant women with history of spontaneous singleton pregnancy under the brand name Makena in the United States. While the primary mode of action is through agonism of progesterone receptor (PR), 17-OHPC has also been identified as a selective modulator of the glucocorticoid receptor (GR). 17-OHPC exhibits selective modulation of endogenous GR target genes, it down-regulates the expressions of *GILZ* and *FKBP5* while showing no impact on *ENaC* (Gerber et al., 2009). Given the importance of the PR and GR in regulating immune responses and inflammation, agonism of PR and selective modulation of GR by 17-OHPC provide a molecular basis for its potential anti-inflammatory and immuno-modulatory activity. Indeed, in a mouse model of lipopolysaccharide (LPS)-induced intrauterine inflammation, maternal serum IL-6 level was significantly reduced after 17-OHPC treatment (Elovitz and Mrinalini, 2006). Using the same mouse model, 17-OHPC was found to reduce CXCL9 and CXCL10 levels in placenta, leading to the modulation of T cell-mediated immunity (Novak et al., 2018). A recent clinical study observed increased levels of IL-1α, IL-1β, IL-2, and IL-13 in the vaginal washings of women at risk for preterm birth, which appeared to be modified to levels similar to healthy controls following the administration of 17-OHPC (Garry et al., 2018). Additionally, both LPS- and lipoteichoic acid (LTA)-induced IL-6 production was significantly less in peripheral blood mononuclear cells (PBMCs) from pregnant women receiving weekly 17-OHPC injection, compared with that in PBMCs from gestational age-matched control subjects (Foglia et al., 2010). Together, these findings demonstrate that 17-OHPC suppresses the production of certain cytokines while treating preterm birth. However, the pharmacological potential of 17-OHPC on the mitigation of the production of multiple cytokines remains largely unknown.

In this regard, the current work studied the inhibitory effect of 17-OHPC on cytokine production both *in vitro* and *in vivo* as well as possible underlying mechanisms, with an aim to provide pharmacological evidence to support the potential application of 17-OHPC in the treatment of CRS associated with COVID-19.

## Materials and Methods

### Materials

17-OHPC was synthesized by Symbiotec Pharmalab (Lot # ZHPCy19003) and provided by Evergreen Therapeutics, Inc. for the studies, castor oil (Sigma-Aldrich, #C9606) and 50% ethanol in PBS were used as the vehicles for *in vivo* and *in vitro* experiments, respectively. Muromonab-CD3 (OKT3) and anti-CD28 antibody were from Takara (#T210) and Sigma-Aldrich (# 217669), respectively. Phytohemagglutinin (PHA) was obtained from Sigma-Aldrich (# 11249738001). BrdU assay kit was from Cell Signaling Technology (# 6813). The antibodies used in this study included anti-phospho-IκBα (# 2859), anti-IκBα (# 9242), anti-NF-κB p65 (# 8242), anti-HDAC1 (# 5356) from Cell Signaling Technology, and anti-GAPDH (# ab9485) from Abcam. Unless otherwise specified, all cell culture reagents were purchased from Sigma-Aldrich or Life Technologies.

### *In vivo* Cytokine Release Assay in PBMC-Engrafted Immunodeficient Mice

The animal work was conducted at The Jackson Laboratory (Sacramento, CA) in compliance with approval of the Institutional Animal Care and Use Committee (Jackson Laboratory protocol # 12027). Seven-week female NOD scid gamma (NSG^TM^)-(K^b^D^b^)^null^ (IA)^null^ (Stock # 025216) mice were housed in individually ventilated polysulfone cages with HEPA filtered air at a density of up to 5 mice per cage. The animal room was lighted entirely with artificial fluorescent lighting, with a controlled 12-h light/dark cycle. The normal temperature and relative humidity ranges in the animal rooms were 22–26°C and 30–70%, respectively. Filtered tap water, acidified to a pH of 2.5–3.0, and standard rodent chow were provided *ad libitum*.

**FIGURE 1:**
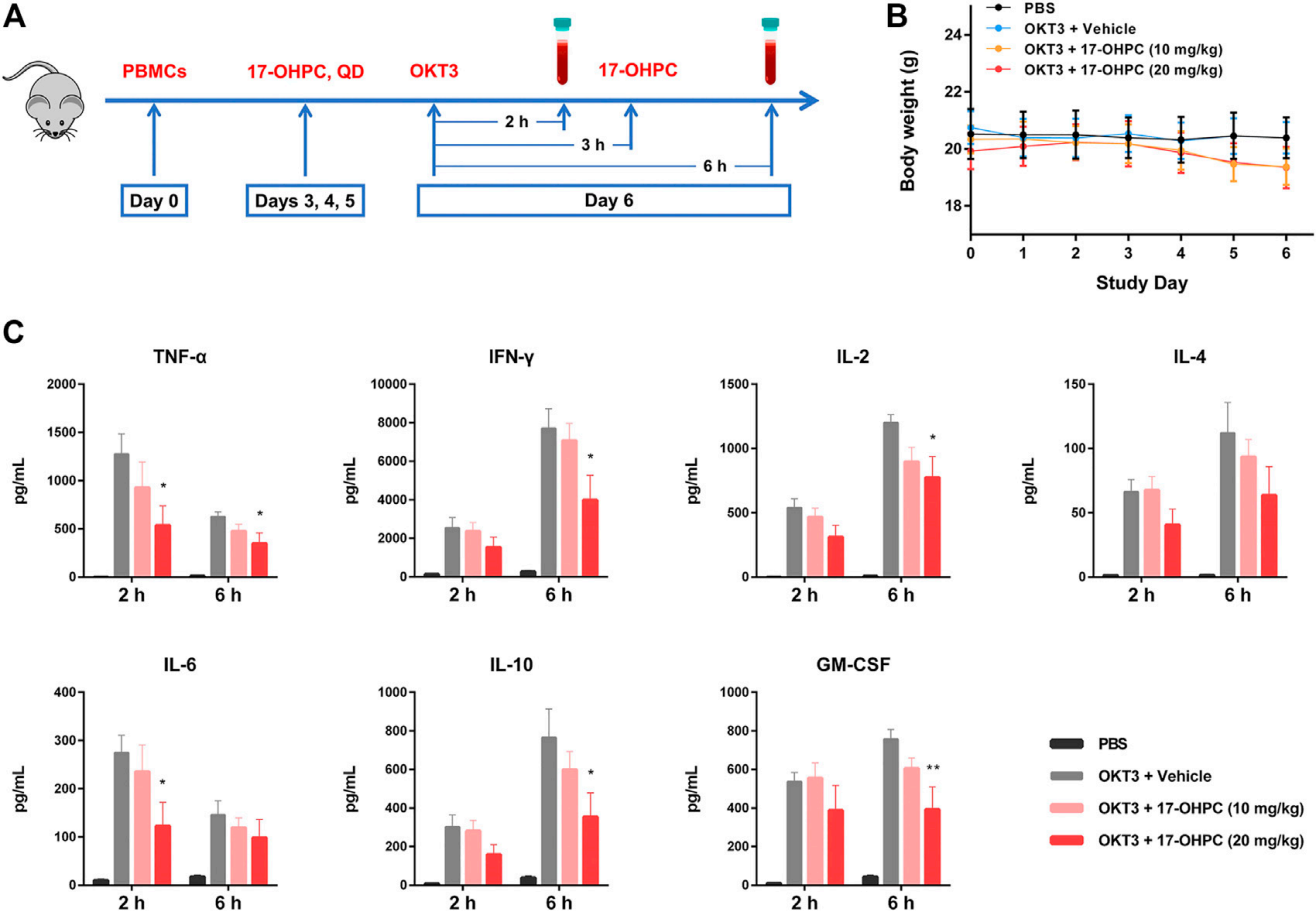
17-OHPC inhibited OKT3-stimulated cytokine production in human PBMC-engrafted immunodeficient mice. **(A)** Experimental design of *in vivo* cytokine release assay in human PBMC-engrafted immunodeficient mice. On study Day 0, mice were irradiated (100 cGy) for at least 4 h and injected intravenously with 15 × 10^6^ human PBMCs. 17-OHPC was administrated once daily starting from Day 3 till Day 6. To stimulate the cytokine release, mice were injected intravenously with OKT3 (0.5 mg/kg) on Day 6, and the blood samples were collected via retro-orbital bleeding at 2 and 6 h after OKT3 administration for further analysis. **(B)** The body weights of human PBMC-engrafted immunodeficient mice in the *in vivo* cytokine release assay. **(C)** The levels of human cytokines including TNF-α, IFN-γ, IL-2, IL-4, IL-6, IL-10, and GM-CSF in blood samples from PBMC-engrafted mice were analyzed using a BD Cytometric Bead Array Human Th1/Th2 Cytokine kit II (Mean ± SEM, *n* = 5; *p < 0.05, **p < 0.01, compared with OKT3 plus vehicle group).

The animal study design was shown in Figure 1A. Briefly, on study Day 0, mice were irradiated and injected intravenously with 15 × 10^6^ human PBMCs (Donor # 9636). Mice were randomized to the following groups and dosed accordingly starting from Day 3: 1) PBS; 2) OKT3 plus vehicle; 3) OKT3 plus 17-OHPC (10 mg/kg); 4) OKT3 plus 17-OHPC (20 mg/kg). To stimulate the cytokine release, mice were injected intravenously with OKT3 (0.5 mg/kg) on Day 6, and the blood samples were collected via retro-orbital bleeding at 2 and 6 h after OKT3 administration. The body weights of mice were recorded everyday throughout the study. The levels of human cytokines including TNF-α, IFN-γ, IL-2, IL-6, IL-10, and GM-CSF in blood samples were analyzed using a BD Cytometric Bead Array Human Th1/Th2 Cytokine kit II (BD-Biosciences).

### Cell Culture and Proliferation

Cryopreserved human PBMCs were pooled from three donors: human donor 4 (Lot # 98), donor 5 (Lot # 99) and donor 6 (Lot # 101) at Eurofins Discovery (St. Charles, MO). The cells were cultured in RPMI1640 medium, supplemented with 10% heat-inactivated FBS, 2 mM L-glutamine, 100 U/ml penicillin and 100 μg/ml streptomycin at 37°C in a humidified atmosphere with 5% CO_2_. The proliferation of PBMCs were measured using BrdU assay according to the manufacturer’s protocol.

### *In vitro* Cytokine Release Assay in Human PBMCs

Three stimulators including OKT3, anti-CD28 antibody, and PHA were individually used to stimulate the cytokine production in human PBMCs. Prior to the assay using anti-CD28 antibody, high binding plates were coated with anti-CD28 (ANC28.1/5D10, 2 μg/well) in PBS and incubated overnight in a biosafety cabinet with the lid open and immobilized by air-dry.

PBMCs were seeded into 96-well polypropylene plates at a density of 1.2–2.0 × 10^5^ cells/well. After pre-incubation at 37°C for 1 h, 17-OHPC at different concentrations (0.1, 1, and 10 μM) was added to the plates and incubated with the cells for 16 h. Afterwards, for assay stimulated by anti-CD28 antibody, the cells were transferred to the anti-CD28 coated plates and incubated for additional 48 h; for assays stimulated by OKT3 or PHA, OKT3 (15 μg/ml) or PHA (10 μg/ml), each was added to the wells and incubated for additional 48 h. Following the incubation, plates were centrifuged, and cell culture supernatants were collected and stored at −80°C until further analysis. The levels of TNF-α, IFN-γ, IL-1β, IL-2, IL-6, and IL-10 in cell culture supernatants were determined by Luminex methodology, per the manufacturer’s protocol, using the Human Cytokine/Chemokine Magnetic bead panel from Millipore Sigma (# HCYTOMAG-60K) with a standard range of 3.2, 16, 80, 400, 2,000, and 10,000 pg/ml.

### Reverse Transcription and Polymerase Chain Reaction

The mRNA levels of cytokines including TNF-α, IFN-γ, IL-1β, IL-2, IL-6, and IL-10 in human PBMCs were determined by quantitative real-time PCR. PBMCs were seeded into 12-well plates at a density of 1.0 × 10^6^ cells/mL and treated as the study design of *in vitro* OKT3-stimulated cytokine release assay. Following the incubation, cells were harvested, the total RNA was isolated using Trizol reagent from Invitrogen and the concentration was measured by a Thermo NanoDrop2000c Spectrophotometer at 260 nm. The same amount of total RNA (1 μg) was used to synthesize cDNA by reverse transcription using the High-Capacity cDNA Reverse Transcription Kit (Applied Biosystems). Real-time PCR was performed at 95°C for 30 s, followed by 40 cycles of 95°C for 5 s and 60°C for 30 s. The expression levels of cytokines were normalized to GAPDH.

### Protein Expression by Western Blot Analysis

The protein expression levels in human PBMCs were examined using western blot analysis. PBMCs were seeded into 6-well plates at a density of 1.0 × 10^6^ cells/ml and treated as the study design of *in vitro* OKT3-stimulated cytokine release assay. Following the incubation, the cells were harvested, and protein concentration was determined using BCA protein assay kit (Pierce Biotechnology). Equal amounts of protein were resolved by SDS-PAGE and transferred onto PVDF membranes, afterwards, the membranes were incubated at 4°C overnight with different antibodies diluted in 5% BSA in washing buffer. Following, the membranes were incubated with HRP-conjugated secondary antibodies at room temperature for 2 h. Chemiluminescent signals were then developed with LumiGLO reagent and Peroxide (Cell Signaling Technology, # 7003) and detected by the Bio-Rad ChemiDoc XRS gel documentation system.

### Statistical Analysis

Statistical analysis of the results was performed using GraphPad Prism 6.0 software. All the assays were performed in triplicates, data were expressed as mean ± standard error of mean (SEM). The significance of difference between groups was estimated by one-way analysis of variance (ANOVA) followed by Dunnett’s multiple comparisons test, *p* < 0.05 indicated statistical significance.

## Results

### 17-OHPC Inhibited OKT3-Stimulated Cytokine Production in Human PBMC-Engrafted Immunodeficient Mice

OKT3 is a murine monoclonal antibody that binds to CD3 on the surface of T cells, it can induce the activation of T cells before exerting immunosuppressive effects (Bugelski et al., 2009). OKT3-stimulated cytokine production in human PBMC-engrafted immunodeficient mice is a well-established animal model to study CRS (Ye et al., 2020). During this study, all the mice were monitored daily for overall health condition and body weights were recorded, no significant changes of body weights were found after PBMCs injection and 17-OHPC treatments (Figure 1B).

In Figure 1C, when compared with the non-stimulated group, the blood levels of human cytokines including TNF-α, IFN-γ, IL-2, IL-4, IL-6, IL-10, and GM-CSF were elevated remarkably both 2 and 6 h after OKT3 administration, suggesting the successful stimulation of cytokine production in mice. Specifically, at 2-h timepoint, IFN-γ showed the highest concentration, followed by TNF-α, IL-2, GM-CSF, IL-10, IL-6, and IL-4; whereas the order of concentrations from highest to lowest was IFN-γ, IL-2, IL-10, GM-CSF, TNF-α, IL-6, and IL-4 at 6-h timepoint. Interestingly, the blood level of each cytokine at 6-h timepoint was higher than that of 2-h timepoint, except TNF-α and IL-6, which showed declined concentrations over time. Importantly, 17-OHPC treatment exhibited dose-dependent inhibition of the production of all cytokines measured in this study. While compared with the OKT3-stimulated group, a statistically significant difference was found after 20 mg/kg 17-OHPC treatment which decreased TNF-α and IL-6 at 2-h timepoint, as well as decreased TNF-α, IFN-γ, IL-2, IL-10, and GM-CSF at 6-h timepoint. These findings demonstrated the *in vivo* inhibition of cytokine production by 17-OHPC in a humanized mouse model of CRS, with a good safety profile.

### 17-OHPC Inhibited OKT3-Stimulated Cytokine Production in Human PBMCs

**FIGURE 2:**
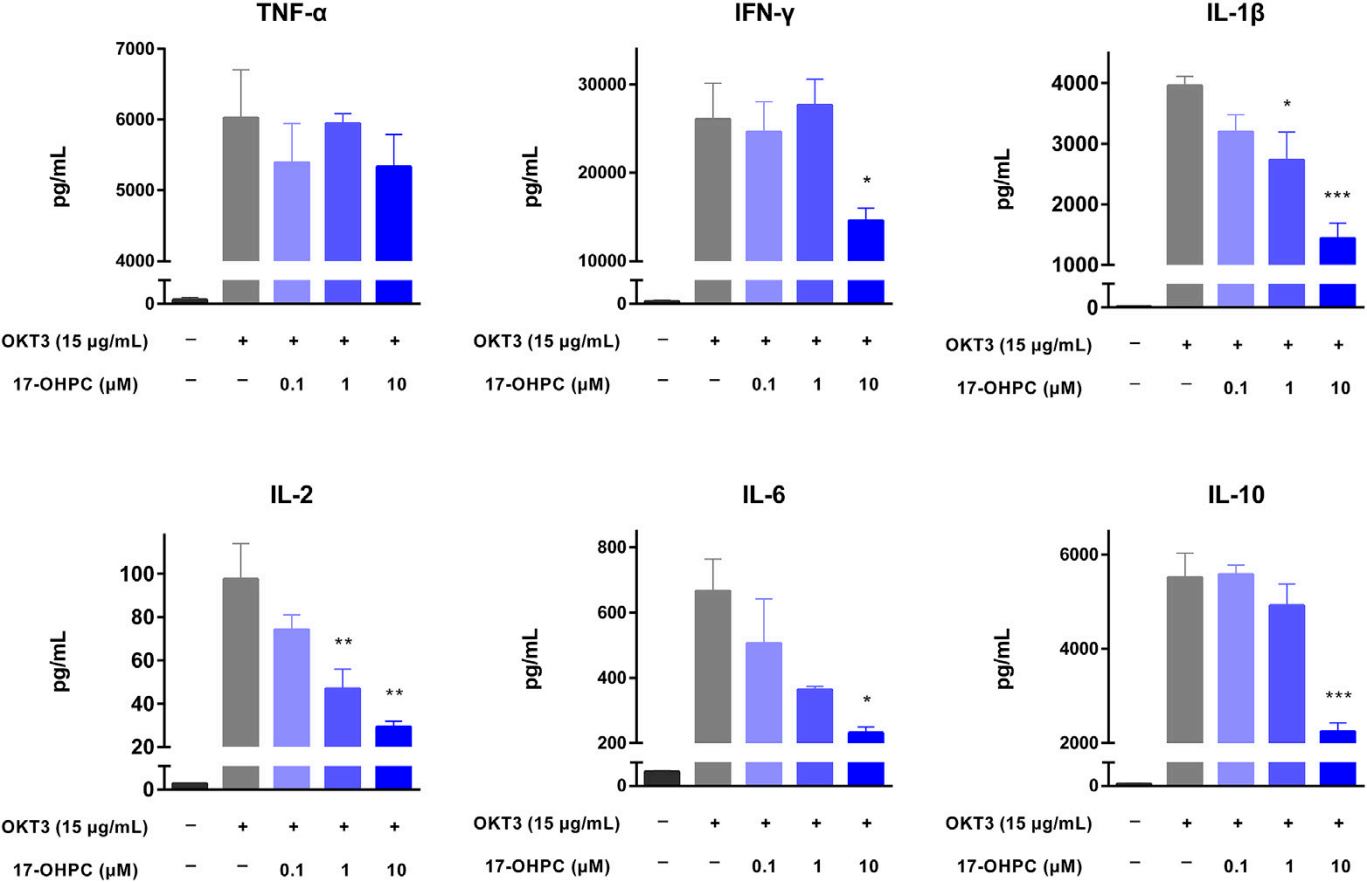
17-OHPC inhibited OKT3-stimulated cytokine production in human PBMCs. PBMCs were seeded into 96-well polypropylene plates at a density of 1.2–2.0 × 10^5^ cells/well. After pre-incubation at 37°C for 1 h, 17-OHPC at different concentrations (0.1, 1, and 10 μM) was added to the plates and incubated with the cells for 16 h. Afterwards, OKT3 (15 μg/ml) was added to the wells and incubated for additional 48 h. Following the incubation, plates were centrifuged, and cell culture supernatants were collected and stored at −80°C until further analysis. The levels of TNF-α, IFN-γ, IL-1β, IL-2, IL-6, and IL-10 in cell culture supernatants were determined using the Human Cytokine/Chemokine Magnetic bead panel from Millipore Sigma (Mean ± SEM, *n* = 3; *p < 0.05, **p < 0.01, and ***p < 0.001, compared with OKT3 plus vehicle group).

The inhibition of cytokine production by 17-OHPC was also tested using a human PBMCs-based *in vitro* cytokine release assay. Prior to the assay, the proliferation of PBMCs was measured, and no significant changes were found after 17-OHPC treatments up to 10 μM (data not shown). When compared with the non-stimulated cells, OKT3 administration successfully stimulated a robust production of TNF-α, IFN-γ, IL-1β, IL-2, IL-6, and IL-10 in PBMCs pooled from three healthy donors, as shown in Figure 2. Upon OKT3 stimulation, IFN-γ showed the highest absolute level, followed by TNF-α, IL-10, IL-1β, IL-6, and IL-2. Similar to the *in vivo* findings, 17-OHPC significantly inhibited OKT3-stimulated production of IFN-γ, IL-1β, IL-2, IL-6, and IL-10 in a concentration-dependent manner. The only exception was TNF-α, of which the level was slightly decreased by 17-OHPC at highest concentration.

### 17-OHPC Inhibited Cytokine Production in Human PBMCs Stimulated by Anti-CD28 Antibody/PHA

To further study the inhibition of cytokine production by 17-OHPC, *in vitro* cytokine release assays were conducted in human PBMCs exposed to additional stimulators including anti-CD28 antibody and PHA. The proliferation of PBMCs was not significantly changed after treatments with 17-OHPC up to 10 μM (data not shown).

**FIGURE 3:**
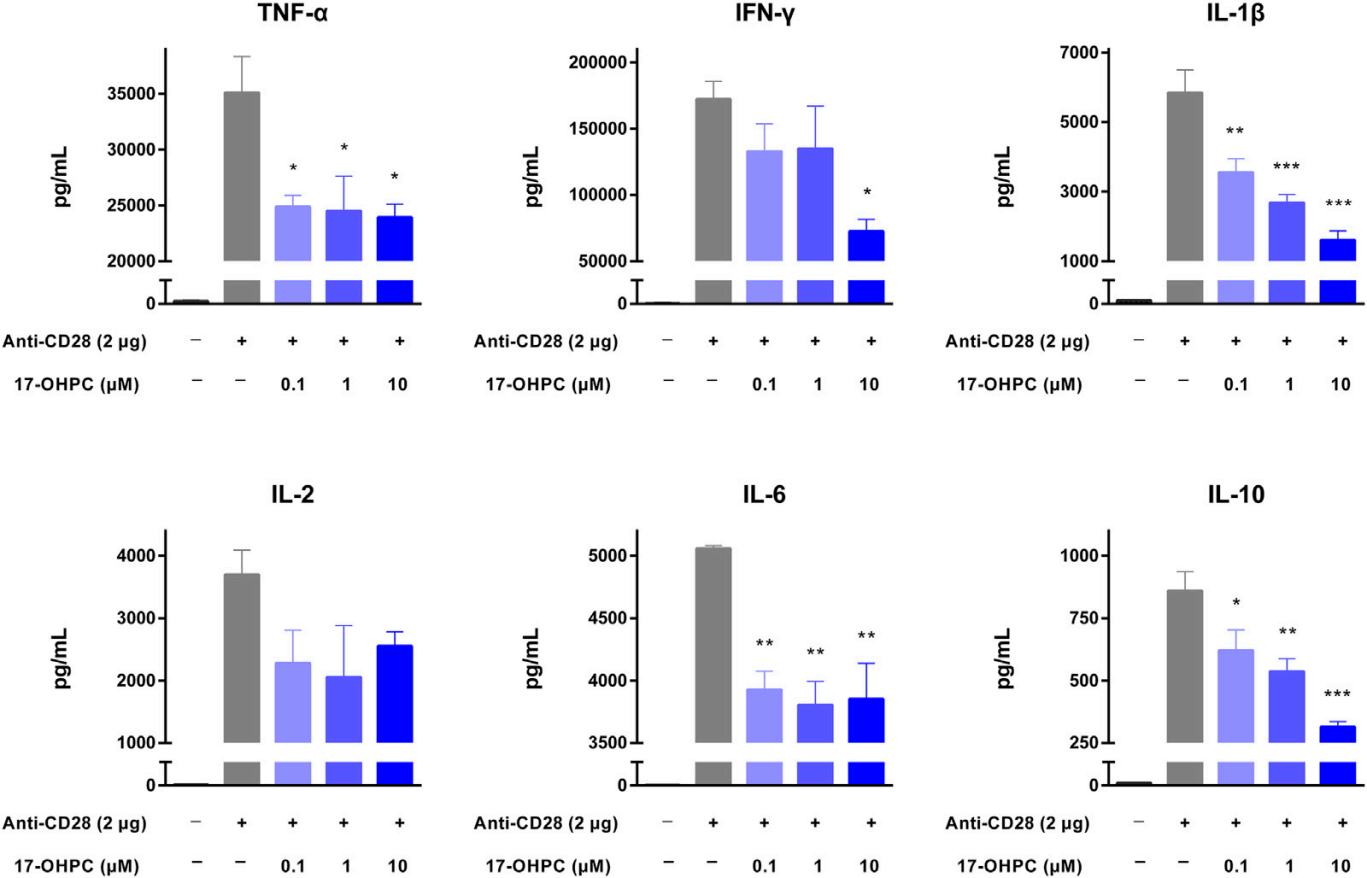
17-OHPC inhibited anti-CD28-stimulated cytokine production in human PBMCs. Prior to the assay using anti-CD28 antibody, high binding plates were coated with anti-CD28 (ANC28.1/5D10, 2 μg/well) in PBS and incubated overnight in a biosafety cabinet with lid open and immobilized by air-dry. PBMCs were seeded into 96-well polypropylene plates at a density of 1.2–2.0 × 10^5^ cells/well. After pre-incubation at 37°C for 1 h, 17-OHPC at different concentrations (0.1, 1, and 10 μM) was added to the plates and incubated with the cells for 16 h. Afterwards, the cells were transferred to the anti-CD28 coated plates and incubated for additional 48 h. Following the incubation, plates were centrifuged, and cell culture supernatants were collected and stored at −80°C until further analysis. The levels of TNF-α, IFN-γ, IL-1β, IL-2, IL-6, and IL-10 in cell culture supernatants were determined using the Human Cytokine/Chemokine Magnetic bead panel from Millipore Sigma (Mean ± SEM, *n* = 3; *p < 0.05, **p < 0.01, and ***p < 0.001, compared with OKT3 plus vehicle group).

**FIGURE 4:**
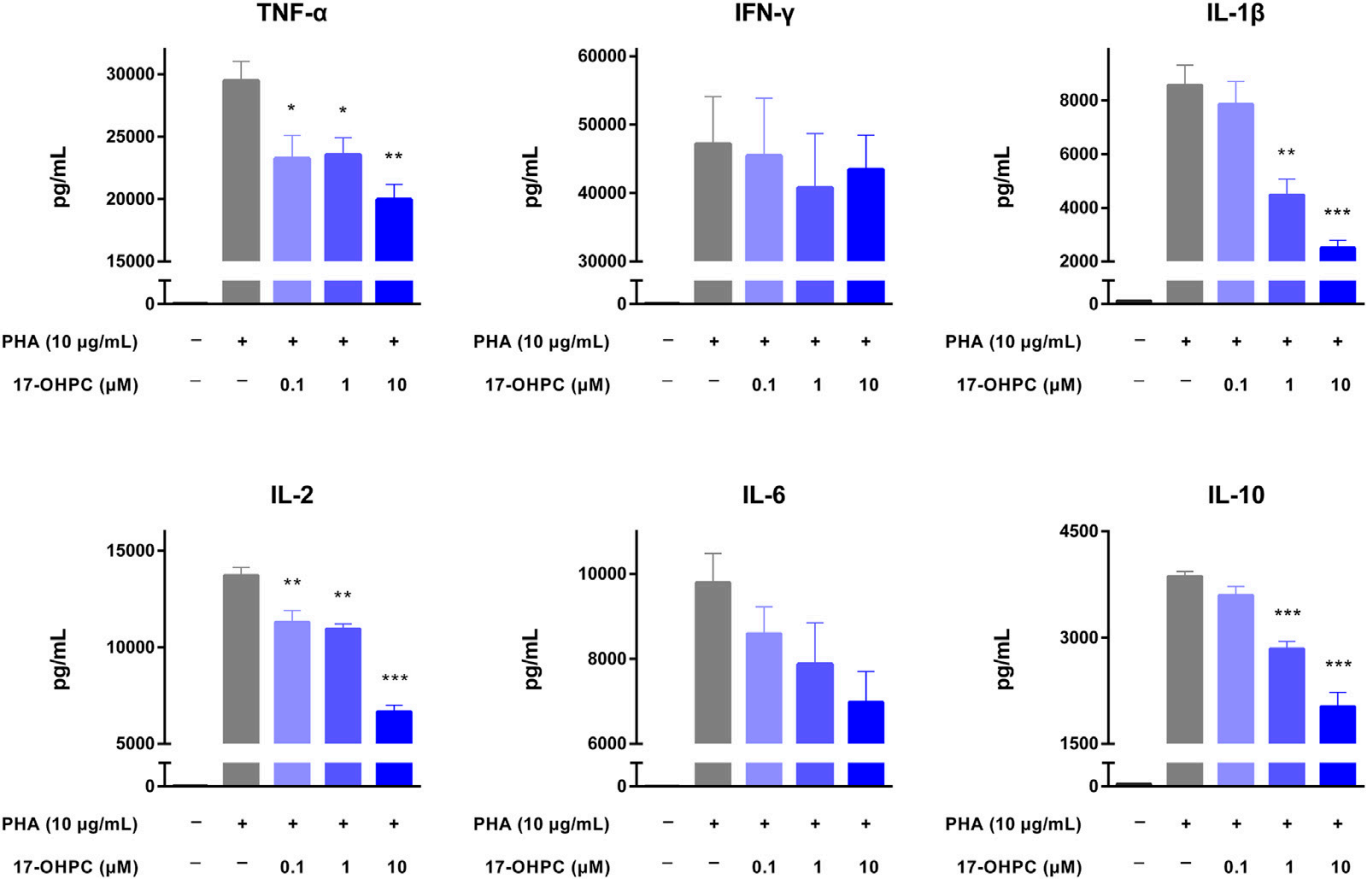
17-OHPC inhibited PHA-stimulated cytokine production in human PBMCs. PBMCs were seeded into 96-well polypropylene plates at a density of 1.2–2.0 × 10^5^ cells/well. After pre-incubation at 37°C for 1 h, 17-OHPC at different concentrations (0.1, 1, and 10 μM) was added to the plates and incubated with the cells for 16 h. Afterwards, PHA (10 μg/ml) was added to the wells and incubated for additional 48 h. Following the incubation, plates were centrifuged, and cell culture supernatants were collected and stored at −80°C until further analysis. The levels of TNF-α, IFN-γ, IL-1β, IL-2, IL-6, and IL-10 in cell culture supernatants were determined using the Human Cytokine/Chemokine Magnetic bead panel from Millipore Sigma (Mean ± SEM, *n* = 3; *p < 0.05, **p < 0.01, and ***p < 0.001, compared with OKT3 plus vehicle group).

Anti-CD28 antibody is an agonistic antibody to the costimulatory molecule CD28 present on T cells (Beyersdorf et al., 2005). Upon anti-CD28 antibody stimulation, as shown in Figure 3, there were remarkable increases in the levels of all cytokines measured. In terms of the absolute values, IFN-γ was still the highest, followed by TNF-α, IL-1β, IL-6, IL-2, and IL-10. 17-OHPC treatment decreased the levels of those cytokines significantly, apart from IL-2 which had an overall decreased trend (not significantly). PHA is a mitogen targeting T cells. Without antigen specificity for cell activation, stimulation by PHA mainly reflects the overall immune response of cells and individual donor variations (Lin et al., 2018). As shown in Figure 4, PHA also led to significant induction of cytokine release in human PBMCs, the order of absolute levels from highest to lowest was IFN-γ, TNF-α, IL-2, IL-6, IL-1β, and IL-10. 17-OHPC showed concentration-dependent reduction in the levels of TNF-α, IL-2, IL-1β, and IL-10. An overall decreased trend of IL-6 was also found after 17-OHPC treatment, despite no statistical significance. Despite that IFN-γ showed the most robust induction upon PHA stimulation, no significant change in its level was observed even after 17-OHPC treatment at the highest concentration (10 μM). These findings demonstrated the profound capability of 17-OHPC in the suppression of cytokine production caused by multiple stimulators.

### 17-OHPC Decreased mRNA Expression of Cytokines in Human PBMCs Stimulated by OKT3

**FIGURE 5:**
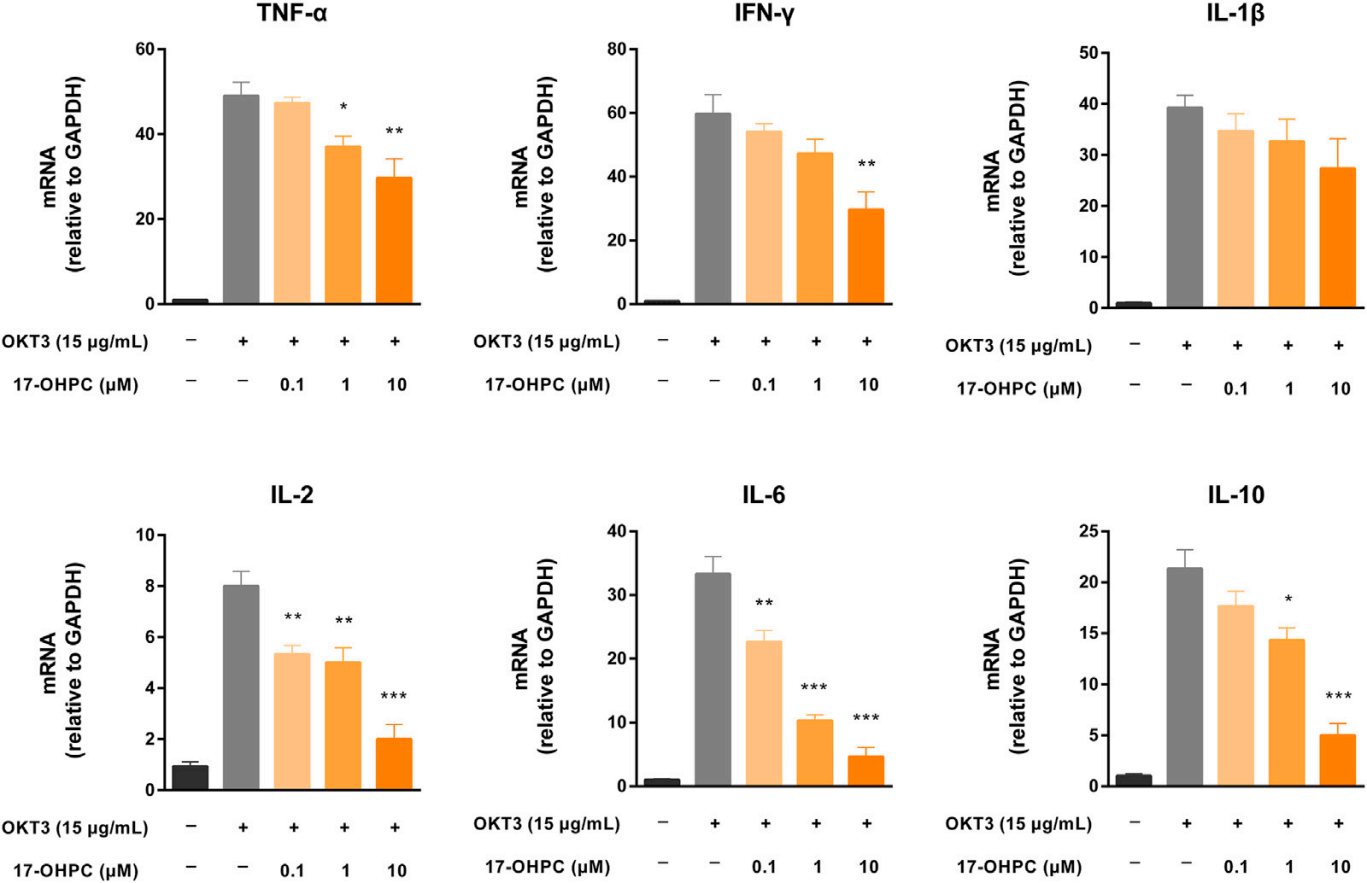
17-OHPC decreased mRNA expression of cytokines in human PBMCs stimulated by OKT3. PBMCs were seeded into 12-well plates at a density of 1.0 × 10^6^ cells/ml. After pre-incubation at 37°C for 1 h, 17-OHPC at different concentrations (0.1, 1, and 10 μM) was added to the plates and incubated with the cells for 16 h. Afterwards, OKT3 (15 μg/ml) was added to the wells and incubated for additional 48 h. Following the incubation, cells were harvested and total RNA was isolated. The mRNA levels of TNF-α, IFN-γ, IL-1β, IL-2, IL-6, and IL-10 were measured by PCR (Mean ± SEM, n = 3; *p < 0.05, **p < 0.01, and ***p < 0.001, compared with OKT3 plus vehicle group).

Given previous successful demonstration of the pharmacological activity of 17-OHPC to reduce cytokine production stimulated by OKT3 both *in vitro* and *in vivo*, the mRNA expression levels of cytokines including TNF-α, IFN-γ, IL-1β, IL-2, IL-6, and IL-10 were measured in human PBMCs after OKT3 and 17-OHPC treatments. Consistent with the cytokine levels in culture medium, OKT3 stimulation resulted in significant up-regulation of the mRNA expression of these cytokines in PBMCs (Figure 5). The highest to lowest mRNA fold change, normalized to GAPDH, was IFN-γ, TNF-α, IL-1β, IL-6, IL-10, and IL-2, similar to the order of the cytokine levels in culture medium. 17-OHPC, as expected, showed concentration-dependent inhibition on the mRNA expression levels of these cytokines.

However, minor discrepancies were noticed between the mRNA expression levels and medium cytokine levels of TNF-α and IL-1β. 17-OHPC exhibited statistically significant reduction of the mRNA expression of TNF-α, while showing no significant effect on its level in culture medium. On the other hand, despite that 17-OHPC showed no significant effect on the mRNA expression of IL-1β, the level of this cytokine showed statistically significant decrease after 17-OHPC treatment. Measurements of the mRNA expression of cytokines in PBMCs and their levels in culture medium at the same timepoint after 17-OHPC exposure may cause such discrepancies. Besides, different observations of TNF-α and IL-1β were speculated to be related to their distinct production kinetics in PBMCs.

### 17-OHPC Suppressed OKT3-Stimulated NF-κB Activation in Human PBMCs

**FIGURE 6:**
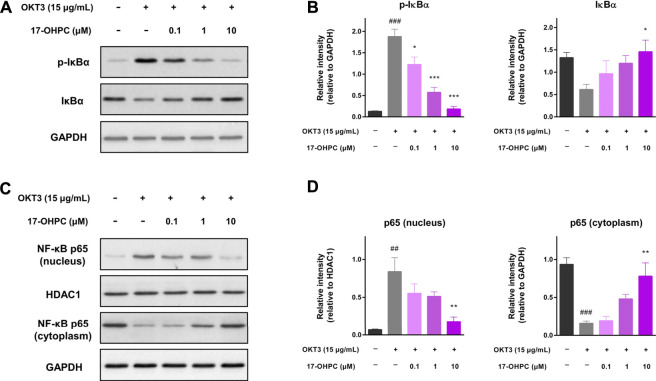
Suppression of OKT3-stimulated NF-κB activation by 17-OHPC in human PBMCs. **(A)** Representative image of the western blot of p-IκBα and IκBα in human PBMCs, showing that 17-OHPC reversed the phosphorylation of IκBα induced by OKT3. **(B)** Statistical analysis of the relative intensities of the western blot bands of p-IκBα and IκBα. (Mean ± SEM, *n* = 3; ###*p* < 0.001, compared with non-stimulated group; *p < 0.05, ***p < 0.001, compared with OKT3 plus vehicle group). **(C)** Representative image of the western blot of NF-κB p65 in nucleus and cytoplasm of human PBMCs, showing that 17-OHPC blocked OKT3-stimulated translocation of NF-κB p65 from cytoplasm into nucleus. **(D)** Statistical analysis of the relative intensities of the western blot bands of NF-κB p65 in nucleus and cytoplasm. (Mean ± SEM, *n* = 3; ##*p* < 0.05, ###*p* < 0.001, compared with non-stimulated group; **p < 0.01, compared with OKT3 plus vehicle group). PBMCs were seeded into 6-well plates at a density of 1.0 × 10^6^ cells/ml. After pre-incubation at 37°C for 1 h, 17-OHPC at different concentrations (0.1, 1, and 10 μM) was added to the plates and incubated with the cells for 16 h. Afterwards, OKT3 (15 μg/ml) was added to the wells and incubated for additional 48 h. Following the incubation, cells were harvested and proteins were extracted for western blot analysis.

The possible molecular mechanisms involved in the inhibition of OKT3-stimulated cytokine production by 17-OHPC were explored by measuring the levels of NF-κB-associated proteins in human PBMCs, given the pivotal role of NF-κB activation in inducing the expression of various pro-inflammatory genes. As shown in Figure 6A, when compared with the non-stimulated cells, there was a significant phosphorylation of IκBα, the protein that suppresses NF-κB activation, and in OKT3 treated cells. Significant reduction of total IκBα expression level was also observed in PBMCs after OKT3 stimulation. However, co-treatment of 17-OHPC reversed the phosphorylation and degradation of IκBα caused by OKT3 stimulation, in a concentration-dependent manner. The inhibitory effects of 17-OHPC on IκBα phosphorylation were statistically significant at 0.1–10 μM, while only 10 μM 17-OHPC showed statistical significance in reversing IκBα degradation (Figure 6B). Next, the cytoplasmic and nuclear levels of NF-κB p65 were measured, OKT3 stimulation led to significant increase of nuclear p65 and decrease of cytoplasmic p65, demonstrating the translocation of NF-κB p65 from cytoplasm into nucleus, and as a result of the phosphorylation and degradation of IκBα. Further treatment with 17-OHPC blocked the translocation of NF-κB p65 concentration-dependently, with statistical significance found in 10 μM group (Figures 6C,D). Those findings demonstrated that inhibition of OKT3-stimulated cytokine production by 17-OHPC in PBMCs was likely associated with its suppression of NF-κB activation.

## Discussion

Despite no difference in the incidence of COVID-19 between male and female patients, a meta-analysis of 3,111,714 reported global cases has identified the male sex as a risk factor for disease severity and mortality (Gerber et al., 2009). Such gender-based bias was speculated to be associated with the differences in sex hormones, cytokine levels, and immune system between sexes, thus providing insights into the potential use of female sex hormones in treating COVID-19. Herein, we reported the activity of a progestogen 17-OHPC in inhibiting CRS, one of the leading causes of severity and mortality associated with COVID-19.17-OHPC inhibited cytokine production both *in vivo* and *in vitro*, such pharmacological activity is potentially attributed to its suppression of NF-κB activation in immune cells.

CRS is a dynamic process that involves continuous changes in circulating levels of various inflammatory cytokines over time. Viral infection by SARS-CoV-2 initially activates innate immune cells, thus recruiting them to the sites of infection and producing pro-inflammatory cytokines including IL-6, IL-12, and TNF-α, etc (Mangalmurti and Hunter, 2020). Following activation of innate immune system, the adaptive immune cells become involved leading to sustained production of inflammatory cytokines, particularly IFN-γ, IL-2, IL-10, TNF-α, and GM-CSF, etc (Yang et al., 2021). In our *in vivo* CRS model, TNF-α and IL-6 showed a rapid and remarkable increase within 2 h after OKT3 stimulation, their levels significantly declined at 6-h timepoint; while other cytokines highly associated with adaptive immune response showed sustained increase within 6 h after stimulation (Figure 1). The difference regarding kinetic changes of those circulating cytokines might reflect the initial innate immune response and subsequent activation of adaptive immune system in this animal model. Interestingly, the level of IL-10, an anti-inflammatory cytokine, was also increased in our *in vivo* and *in vitro* models of cytokine production. This is consistent with the clinical observations which showed elevated circulating levels of IL-10 in patients with CRS associated with COVID-19, TGN1412, and CAT-T cell therapy (Suntharalingam et al., 2006; Morris et al., 2021; Yang et al., 2021). It is speculated that up-regulation of IL-10 is a negative feedback loop in response to excessive immune response, however, such endogenous negative regulators fail to calm aggressive cytokine storm.

17-OHPC is a synthetic progestogen synthesized through the acetylation of 17α-hydroxyprogesterone, the naturally occurring metabolite of progesterone. Of note, progesterone, a natural progestogen, has been reported to exhibit versatile roles in potential therapy of COVID-19. For example, progesterone showed anti-viral activity against SARS-CoV-2 by interfering with the binding of virus to angiotensin-converting enzyme 2 (ACE2), increasing secretory leukocyte protease inhibitor (SLPI), and blocking the up-regulation of transmembrane protease serine 2 (TMPRSS2) by androgens (De Leo et al., 2016; Shah, 2021). Moreover, progesterone can suppress activation of leukocytes and production of inflammatory mediators by binding to PR, which is found in macrophages, dendritic cells, natural killer cells, and T cells (Shah, 2021). Notably, clinical data demonstrated that male patients experiencing moderate to severe COVID-19 and receiving progesterone showed improvements in clinical score status and required fewer days of supplemental oxygen and less hospitalization time, as compared with control subjects (Ghandehari et al., 2021). Collectively, those results provided both basic and clinical evidence supporting agonism of PR as an approach to treat COVID-19. Based on the findings in our *in vivo* and *in vitro* cytokine release assays, 17-OHPC exhibited remarkable inhibition on the production of multiple cytokines, demonstrating its potential in treating CRS in COVID-19 patients. Such inhibitory effects of 17-OHPC were believed to be associated with its binding to PR in immune cells. Additionally, whether 17-OHPC affects the entry of SRAS-CoV-2 into host cells is worthy of further investigation.

Even though the pathogenesis of CRS remains not well understood, the involvement of NF-κB activation in production of various inflammatory cytokines was demonstrated by numerous evidences, supporting NF-κB pathway as a potential target for the treatment of critical stage COVID-19 patients (Kircheis et al., 2020). Consistent with previous reports (Romano et al., 1996), our analysis on the expression levels of key proteins associated with NF-κB also showed robust activation of this signaling pathway upon OKT3 stimulation in human PBMCs (Figure 6). More importantly, 17-OHPC treatment resulted in suppression of OKT3-stimulated NF-κB activation, potentially uncovering a mechanism that contributes to the inhibitory effects of 17-OHPC on cytokine production. Previous literature suggested the mechanisms by which 17-OHPC suppresses NF-κB activation initiated with its binding to PR and/or GR. In fact, agonism of PR by progesterone led to up-regulation of IκBα and suppression of NF-κB activation in human myometrial cells, contributing to the anti-inflammatory activity (Hardy et al., 2006). Activation of GR, on the other hand, has also been proven to inhibit NF-κB signaling pathway in HeLa and other cell lines (De Bosscher et al., 2003; Nelson et al., 2003). Interestingly, glucocorticoids-mediated repression of NF-κB is consistently accompanied by the up-regulation of IκBα in monocytes and lymphocytes, whereas such change of IκBα was not observed in fibroblast and endothelial cells *in vitro* (De Bosscher et al., 2003). Given its agonism of PR and selective modulation of GR, the suppression of NF-κB by 17-OHPC in human PBMCs is likely through a pathway involving activation of PR and/or GR, up-regulation of IκBα and subsequent disruption of p65 translocation into the nucleus. Apart from NF-κB, cytokine production in immune cells also involves the activation of other transcription factors such as nuclear factor of activated T cells (NFAT) and activator protein 1 (AP-1) (Whisler et al., 1996; Jutz et al., 2016). In this regard, studies on NFAT and AP-1 would further improve our understanding on the molecular mechanisms of 17-OHPC to inhibit cytokine production.

As a known sequela to ARDS, the initiation and progression of pulmonary fibrosis is also highly associated with cytokine storm (Rai et al., 2021). Notably, the incidence rate of post-COVID-19 lung fibrosis was estimated to be 2–6% after moderate illness, the prevalence of COVID-19-associated lung fibrosis is 30 times higher than that of idiopathic pulmonary fibrosis (IPF) (Bazdyrev et al., 2021). In addition to the inhibition of cytokine production, agonism of PR also exhibited promising pharmacological activities related to the improvement of lung functions. In a mouse model of airway remodeling induced by ozone, for instance, progesterone was found to decrease collagen deposition in lung tissues, and accompanied by down-regulation of the expression levels of matrix metallopeptidase (MMP)8 and MMP9 (Zhang et al., 2018). Besides, the protein expression of α-smooth muscle action (α-SMA), an indicator of fibrosis, was also reduced after progesterone treatment (Zhang et al., 2018). Using fibroblasts-to-myofibroblasts transition (FMT) model in human fetal lung fibroblast cells MRC-5, we also found that transforming growth factor (TGF)-β-induced up-regulation of α-SMA was significantly reduced by 17-OHPC (unpublished data). Those finding demonstrated the potential of 17-OHPC to prevent airway remodeling and pulmonary fibrosis, with this response expected to provide additional beneficial effects in the inhibition of pulmonary fibrosis and improvement of lung functions in COVID-19 patients.

Lastly, there are a few limitations of the current work: 1) OKT3-stimulated cytokine production in human PBMC-engrafted immunodeficient mice is an effective model to identify inhibitors of CRS (Ye et al., 2020), however, this is not an exact model producing CRS directly associated with coronavirus infection; 2) OKT3, anti-CD28 antibody and PHA are considered typical stimulators of T cells (Ceuppens et al., 1988; Beyersdorf et al., 2005; Bugelski et al., 2009), the effects of 17-OHPC on the activation of other immune cells such as B cells and macrophages are not well studied; 3) activation of NF-κB only represents one of the mechanisms of cytokine production in immune cells, the effects of 17-OHPC on other transcription factors, such as NFAT and AP-1 (Whisler et al., 1996), remains unknown. In this regard, future investigation will include additional efficacy studies in animal models with higher relevance to CRS in COVID-19 patients, *in vivo* and *in vitro* cytokine release assays using additional stimulators including the typical B cell activator LPS (Venkataraman et al., 1999), and possible regulation of NFAT and AP-1 in immune cells by 17-OHPC.

In summary, this work reported both *in vivo* and *in vitro* inhibition of cytokine production by 17-OHPC, presumably by virtue of its suppression of NF-κB activation. These findings provided pharmacological evidences to support the potential application of 17-OHPC in treating CRS associated with COVID-19. Sponsored by Evergreen Therapeutics, Inc., the phase II clinical trial of 17-OHPC is currently in progress (ClinicalTrials.gov Identifier: NCT04561180), with an aim to provide an effective therapy for the treatment of moderate and severe COVID-19 patients.

## Abbreviations

ACE2, angiotensin-converting enzyme 2; AP-1, activator protein 1; ARDS, acute respiratory distress syndrome; CAR-T cell, chimeric antigen receptor-T cell; CCL, chemokine C-C motif ligand; COVID-19, coronavirus disease 2019; CRS, cytokine release syndrome; CXCL, chemokine C-X-C motif ligand; FMT, fibroblasts-to-myofibroblasts transition; GM-CSF, granulocyte-macrophage colony-stimulating factor; GR, glucocorticoid receptor; 17-OHPC, 17α-hydroxyprogesterone caproate; α-SMA, α-smooth muscle action; IFN-γ, interferon-γ; IL, interleukin; NF-κB, nuclear factor-κB; NFAT, nuclear factor of activated T cells; OKT3, muromonab-CD3; PBMC, peripheral blood mononuclear cell; PHA, phytohemagglutinin; PR, progesterone receptor; SARS-CoV-2, severe acute respiratory syndrome coronavirus 2; TNF-α, tumor necrosis factor-α; TGF-β, transforming growth factor-β.

## Data Availability

The raw data supporting the conclusion of this article will be made available by the authors, without undue reservation.
